# The spatial distribution of leprosy in four villages in Bangladesh: An observational study

**DOI:** 10.1186/1471-2334-8-125

**Published:** 2008-09-23

**Authors:** EAJ Fischer, D Pahan, SK Chowdhury, L Oskam, JH Richardus

**Affiliations:** 1 Department of Public Health, Erasmus MC, University Medical Center Rotterdam, Rotterdam, The Netherlands; 2 Rural Health Program, Leprosy Mission Bangladesh, Nilphamari, Bangladesh; 3 KIT Biomedical Research, Royal Tropical Institute, Amsterdam, The Netherlands

## Abstract

**Background:**

There is a higher case-detection rate for leprosy among spatially proximate contacts such as household members and neighbors. Spatial information regarding the clustering of leprosy can be used to improve intervention strategies. Identifying high-risk areas within villages around known cases can be helpful in finding new cases.

**Methods:**

Using geographic information systems, we created digital maps of four villages in a highly endemic area in northwest Bangladesh. The villages were surveyed three times over four years. The spatial pattern of the compounds – a small group of houses – was analyzed, and we looked for spatial clusters of leprosy cases.

**Results:**

The four villages had a total population of 4,123. There were 14 previously treated patients and we identified 19 new leprosy patients during the observation period. However, we found no spatial clusters with a probability significantly different from the null hypothesis of random occurrence.

**Conclusion:**

Spatial analysis at the microlevel of villages in highly endemic areas does not appear to be useful for identifying clusters of patients. The search for clustering should be extended to a higher aggregation level, such as the subdistrict or regional level. Additionally, in highly endemic areas, it appears to be more effective to target complete villages for contact tracing, rather than narrowly defined contact groups such as households.

## Background

Identifying individuals with increased exposure to *Mycobacterium leprae*, the causative agent of leprosy, enhances the possibility of prevention or early diagnosis. Several studies have shown that household members and neighbors have an increased risk of leprosy [1-3], making them desirable targets for interventions such as preventive treatment [2,4]. A study in Indonesia identified spatial clusters of cases on islands with extremely high incidence [1]. Spatial information can be used to improve the discovery of new cases and other interventions in high incidence areas [5].

In the Nilphamari district in Bangladesh, household members and close neighbors have an increased risk of contracting leprosy when compared with neighbors of neighbors and social contacts [2]. However, new cases among neighbors of neighbors and social contacts were still over three times more likely than in the general population [2,6]. Because neighbors of neighbors and social contacts still live near patients [7], exposure to *M. leprae *is likely to cluster at a spatial level smaller than villages. Moet *et al. *[2] have shown that leprosy is aggregated at the household level and for adjacent neighbors, but the extent to which leprosy cases are spatially aggregated within complete villages is not known.

We believe that identifying neighborhoods or areas with many previously undetected cases will improve efforts to find new cases. Here, we report on the spatial distribution of prevalent cases and cases that were found during two follow-up surveys with two-year intervals in four villages within in a highly endemic area. We attempted to identify spatial clusters of leprosy cases within these four villages using a spatial scan statistic [8,9].

## Methods

### Study population and survey

As part of a larger previously conducted study [10], 20 administrative areas were randomly selected from two districts in northwest Bangladesh. The survey started at the northern borders of the areas and included all of the people present until approximately 1,000 people were examined. The groups were surveyed between November 2002 and February 2003. During the survey, people were asked about leprosy symptoms and a body check was performed. Those who were suspected of having leprosy were referred to a senior leprosy control officer and a doctor for confirmation. If the disease was confirmed, regular treatment was offered. The inhabitants who participated in the first survey were visited in the same months in 2004–2005 and 2006–2007, if they still lived in the same area. So that our results may be thoroughly understood, we have provided a summary of our survey methods. A more extensive description of the survey can be found elsewhere [6].

**Figure 1:**
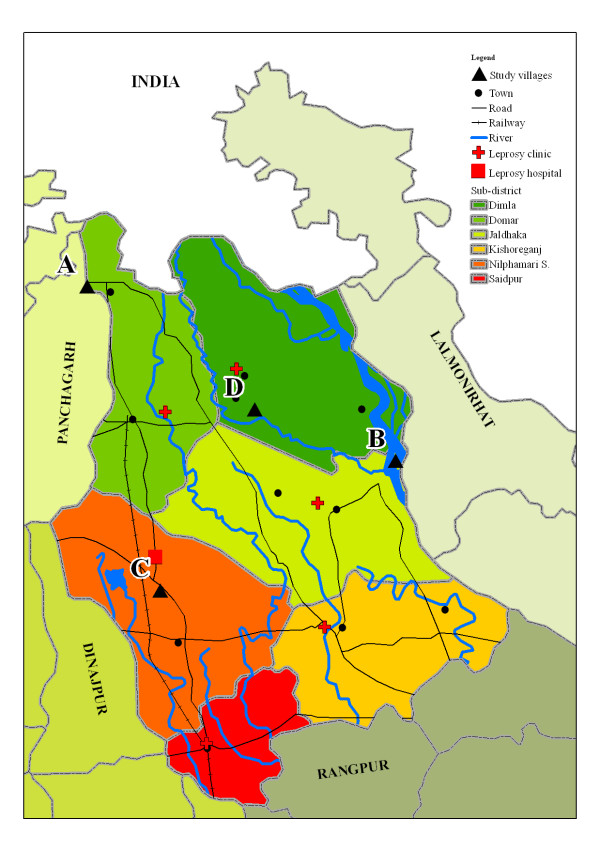
**An overview map of the Nilphamari district showing several geographic features, such as towns, roads and rivers.** The four selected villages are indicated by black triangles.

For the current study, we selected four groups out of the 20 groups, all within the Nilphamari district, because these were easily accessible. An overview map of the Nilphamari indicating the four selected villages is presented in figure 1. We selected the sample populations with the highest number of cases during intake; three of the four selected population samples also had a high prevalence of anti-*M. leprae *IgM antibodies, which is thought to indicate increased exposure [6], most likely leading to an increased incidence of leprosy. Three groups were selected from a rural area and one from an urban area.

### Map preparation and census data

Maps were prepared in January 2006 using handheld global positioning system (GPS) units (Geko 201, Garmin, USA). The maps were drawn in ArcGIS 9.1 (ESRI, USA). Coordinates were collected for the compounds and roads, and for some geographic features such as schools, mosques, and bodies of water. Compounds are small groups of 1 to 10 houses, often inhabited by one family. Digital maps were drawn using these geo-references and hand-drawn maps. The calculated centroids of compounds were used as census points. Participants were attributed to the nearest census points.

We recorded participants' death and migration since the 2003 study intake. If we were able to obtain the information, migrated or deceased people were attributed to the compound in which they lived during intake.

### Statistical analysis

The spatial pattern of the compounds was determined by the average nearest neighbor index (ANNI). An ANNI smaller than 1 indicates a clustered pattern of compounds when compared with a random model [11].

The groups were scanned using spatial scan statistic to detect high prevalence clusters of cases. The scans were performed for purely spatial data, and imposed circular windows with flexible radii on all of the locations in the area. The number of cases within a window was assumed to follow a Poisson distribution under the null hypothesis. For each window, the likelihood was calculated for the observed cases and the expected cases under the null hypothesis. The window with the highest likelihood constituted the most likely cluster. The distribution of the maximum likelihood was determined by many random replications of the dataset under the null hypothesis. The *p *value was then calculated by comparing the rank of the maximum likelihood of the real dataset with the ranks of the maximum likelihoods of the random datasets [8]. The analyses were performed with SatScan version 7.0 [9].

### Ethical clearance

Ethical clearance was obtained from the ethical review committee of the Bangladesh Medical Research Council (reference numbers BMRC/ERC/2001–2004/799 and BMRC/ERC/2004–2007/1397).

## Results

### Area characteristics

Group A lived in an area near the Indian border. The total area of the village was 1.04 km^2^. The village contained two schools for secondary education and a local police headquarters.

Group B was reached by crossing a large river. The east and west borders of the village were delimited by the river embankments. The village contained no brick or concrete buildings, except for a mosque and a primary school. The total area of the village was 1.39 km^2^. It bordered another village directly to the north.

The urban group C was located at the edge of the district capital and contained the largest population of the three groups. This urban ward had an area of only 0.31 km^2^. Most of the compounds were north of an asphalt road leading to the town center. Approximately one-third of the houses were built of brick or concrete. The office building of a large regional nongovernmental organization was located on the south border.

Group D was located near a cluster of shops situated at the crossing of two major roads coming from the district capital and a nearby town. A lake surrounding by marshes bordered the village to the south. At 1.82 km^2^, this village had the largest area of the four groups. The village contained two primary schools, two mosques, and several Hindu shrines.

### Study population

The total study population consisted of 4,123 people. The mean age at intake was 21.8 years. The proportion of children under 15 years was on average 0.54. People who were not at home during intake were not included in the study, which is the most likely explanation for the uneven sex ratios of groups A, C, and D (Table 1). In these groups, males were more likely than females to be at work in another area during the days on which intake took place. The people of group B worked in the fields near their home; thus, males and females were evenly included.

**Table 1:** Study population, demographics, and number of newly detected leprosy cases in four sample populations.

			**Population sample**				
			*A rural*	*B rural*	*C urban*	*D rural*	*Total*
**Population**	Population size at intake		1008	1000	1107	1008	**4123**
	Mean age	Male	20.1	22.2	17.5	21.6	**20.4**
		Female	22.2	21.9	22.5	24.5	**22.8**
		Both	21.4	22.1	20.5	23.3	**21.8**
	Proportion age < 15		0.57	0.55	0.56	0.49	**0.54**
	Sex ratio		0.7	1.0	0.7	0.7	**0.8**

**Village\Ward**	Compounds		219	167	253	253	**892**
	Inhabitants per compound		4.6	6.0	4.4	4.0	**4.6**
	Houses per compound		2.0	2.0	1.5	2.0	**1.9**
	Inhabitants per house		2.3	3.0	2.9	2.0	**2.5**
	Area (in km^2^)		1.04	1.39	0.31	1.82	**4.56**

**Leprosy**	RFT before intake*		3	3	8	0	**14**
	Case at intake		6	0	1	0	**7**
	Case at 1^st ^follow up		3	0	2	1	**6**
	Case at 2^nd ^follow up		1	0	5	0	**6**

*Released from leprosy treatment before the first survey.

The average compound size of 4.6 persons per compound was comparable with the census data on average household size for rural Bangladesh [12]. Compounds comprised 1.9 houses, on average.

At intake, 14 persons were known to have been released from treatment for leprosy prior to the study intake. Furthermore, there were seven newly diagnosed cases of leprosy. Of the seven cases, all had paucibacillary (PB) leprosy. There were no cases of multibacillary (MB) leprosy. After two years, six new cases were detected; one had MB leprosy, and the other five were diagnosed with PB leprosy. Finally, four years after study intake, another six new cases were detected, all of which were PB leprosy. The proportion of PB cases was not unexpected, given that the proportion of PB cases among the total cases detected in this district was approximately 0.8. During surveys, such as the one used in this study, the proportion of PB cases is higher than among voluntarily reported cases, because many less-severe cases can remain otherwise undetected.

After two years, a total of 265 persons were lost to follow-up due to death (37) or migration (228). As far as we could determine either by registration at the clinics or by asking relatives, none of the deceased people had experienced clinical leprosy. One person who had migrated was diagnosed with leprosy at intake in group A, and could be attributed to the compound in which he was living at intake. Thirty persons moved within the areas; none of them had leprosy. At the time of the writing of this report, further details concerning persons lost to follow-up were not yet available.

### Spatial patterns

**Figure 2:**
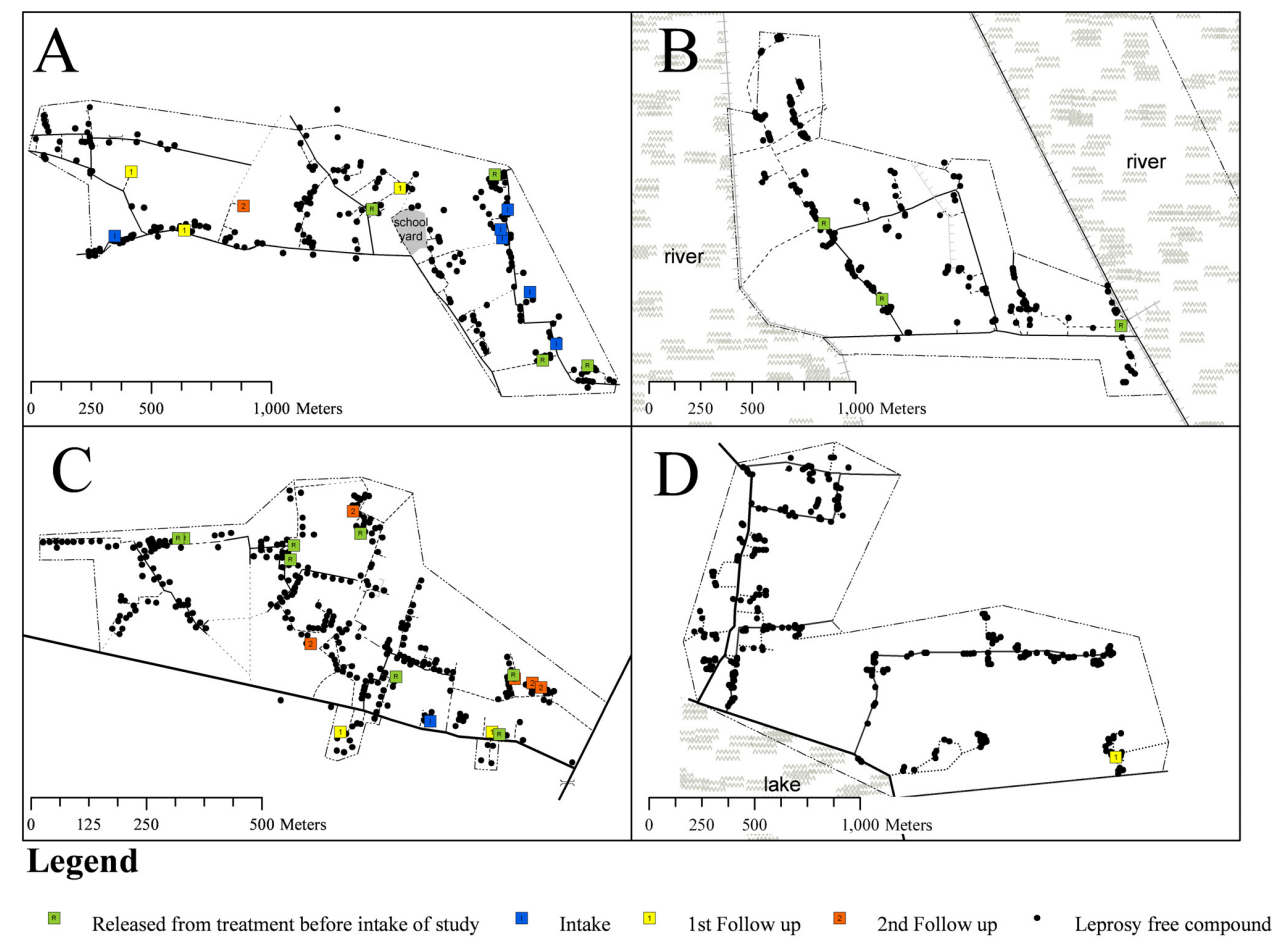
**Newly detected cases by moment of detection (*e.g. *before intake, at intake, first follow up or second follow up) in four sample group areas.** Compounds are depicted by a black dot. The dash-dotted (⋯-) line indicates the village or ward border. Other lines indicate roads, canals and river embankments. Compounds outside these borders are not included in the study, but some are shown on the maps to indicate the closeness of other villages.

Compounds were aggregated in space (Table 2). ANNI ranged between 0.30 and 0.56. Eyeballing of Figure 2 intuitively confirms the aggregated spatial pattern of the compounds, which were positioned in small groups and along the roads.

**Table 2:** Spatial patterns of compounds and new cases in each population

	Clustering of compounds		Clusters of cases							
			All cases		At intake		1^st ^follow up		2^nd ^follow up	
	ANNI^a^	Z-score	LLR^b^	*p*^c^	LLR^b^	*p*^c^	LLR^b^	*p*^c^	LLR^b^	*p*^c^
A^d^	0.42	-17.6	3.2	0.60	5.9	0.13	3.0	0.51	-	-
B^d^	0.30	-17.8	3.4	0.52	-	-	-	-	-	-
C	0.56	-14.7	4.2	0.81	4.5	0.18	1.8	0.65	4.4	0.14
D^d^	0.30	-22.9	-	-	-	-	-	-	-	-

^a ^Average Nearest Neighbor Index.

^b ^Log likelihood ratio.

^c ^Determined by 999 Monte Carlo replications.

^d ^No calculation of log-likelihood ratio – and thus p-value – possible for none or 1 case.

The spatial scan statistic determined the location of the most likely cluster for each area. None of the four clusters were significantly different (*p *< 0.05) from the Poisson model, shown in Table 2.

## Discussion

We could not identify clusters of leprosy at this spatial microlevel of 0.32–1.82 km^2^, an area equivalent to a town ward or village. Thus, either at this level spatial clustering does not occur, or the force with which leprosy clusters is not strong enough to reveal spatial clustering using only a few population samples with a moderate number of cases. One would need to observe many of these areas (villages) to identify a limited number of possible clusters. Both of these explanations call into question the value of attempting to identify leprosy clusters at this level.

Spatial clustering of leprosy has been found on Indonesian islands with extremely high numbers of previously undetected cases [1,13]. The power of the statistical tests for clustering was thus much greater than in our study. Furthermore, the studies by Bakker *et al*. [13] were conducted among populations living on remote islands. These island populations had a contact pattern that differed from our study population. The population in northwest Bangladesh is not confined to an archipelago of small islands, but lives in an easily accessible and densely populated area of the Indian subcontinent.

Although members of the same household as a person with leprosy have a much higher relative risk of contracting leprosy [2], the number of nonhousehold contacts (such as relatives and social contacts) is many times higher than that of household contacts [14]. However, as we have illustrated, we found no clusters of leprosy within the limited number of villages that we observed.

It is not easy to identify clusters of patients using spatial analysis at the microlevel of villages in highly endemic areas, compared with higher levels. In a separate paper, we found spatial clustering at the district level in the same area in Bangladesh [15]. In addition, Moet *et al. *[2] found large differences in previously undetected prevalence in the 20 population samples. Some of these population samples (e.g., group A in this study) had a previously undetected prevalence equal to that of close contacts [2].

## Conclusion

The search for clustering should be extended to higher aggregation levels, such as subdistrict or regional levels. Thus, in highly endemic areas, it appears to be more effective to target complete villages for contact tracing, rather than narrowly defined contact groups such as households.

## Abbreviations

ANNI: average nearest neighbor index; GIS: geographic information system; GPS: global positioning system; MB: multibacillary; PB: paucibacillary.

## Competing interests

The authors declare that they have no competing interests.

## Authors' contributions

EF was involved in all aspects of the research and drafting of this manuscript. DP and SC contributed to the set-up, planning, and conduction of data collection, and commented on the manuscript. LO was involved in the conception and design of the study, and also in drafting of the manuscript. JR was involved in the conception and design of the study, as well as the analysis, and contributed considerably to the drafting of the manuscript.

## Pre-publication history

The pre-publication history for this paper can be accessed here:


